# Racemic crystal structures of A-DNA duplexes

**DOI:** 10.1107/S2059798322003928

**Published:** 2022-05-09

**Authors:** Pradeep K. Mandal, Gavin W. Collie, Brice Kauffmann, Ivan Huc

**Affiliations:** a Université de Bordeaux, CNRS, Bordeaux Institut National Polytechnique, CBMN (UMR 5248), 33600 Pessac, France; b Department of Pharmacy and Center for Integrated Protein Science, Ludwig-Maximilians-University, Munich, Germany; c Université de Bordeaux, CNRS, INSERM, Institut Européen de Chimie et Biologie (UAR3033 and US001), 33600 Pessac, France

**Keywords:** racemic crystallography, DNA, X-ray diffraction, DNA crystallography

## Abstract

The ease with which racemic mixtures crystallize compared with the equivalent chiral systems is routinely taken advantage of to produce crystals of small molecules. However, biological macromolecules such as DNA and proteins are naturally chiral, and thus the limited range of chiral space groups available hampers the crystallization of such molecules. Inspiring work over the past 15 years has shown that racemic mixtures of proteins, which were made possible by impressive advances in protein chemical synthesis, can indeed improve the success rate of protein crystallization experiments. More recently, the racemic crystallization approach was extended to include nucleic acids as a possible aid in the determination of enantiopure DNA crystal structures. Here, findings are reported that suggest that the benefits may extend beyond this. Two racemic crystal structures of the DNA sequence d(CCCGGG) are described which were found to fold into A-form DNA. This form differs from the Z-form DNA conformation adopted by the chiral equivalent in the solid state, suggesting that the use of racemates may also favour the emergence of new conformations. Importantly, the racemic mixture forms interactions in the solid state that differ from the chiral equivalent (including the formation of racemic pseudo-helices), suggesting that the use of racemic DNA mixtures could provide new possibilities for the design of precise self-assembled nanomaterials and nanostructures.

## Abstract 2

Racemic crystallography benefits the identification of a structural form of a DNA sequence that was not previously observed for the enantiopure equivalent.

## 1. Introduction

Racemic mixtures crystallize much more frequently as racemates in non-Sohncke (*i.e.* enantiogenic) space groups than as conglomerates. They also tend to be more amenable to crystallization than the equivalent enantiopure samples (Jacques *et al.*, 1994 ▸; Matthews, 2009 ▸). Crystallography in the chemistry world (*i.e.* small-molecule crystallography) commonly involves racemates or achiral molecules. In contrast, biological macromolecular crystallography involves the study of naturally chiral molecules, such as DNA and proteins, which do not have access to non-Sohncke space groups and are thus limited with respect to packing options (Wukovitz & Yeates, 1995 ▸). However, impressive improvements in chemical peptide (and protein) synthesis over the past twenty years have enabled the generation of racemic protein mixtures, thereby enabling racemic crystallographic methods. Indeed, racemic protein mixtures have been shown to crystallize more readily, invariably forming racemic crystals and enabling successful structure determination when this had failed with the natural l-amino-acid backbone (Yeates & Kent, 2011 ▸). This racemic approach to the crystallization of naturally chiral protein molecules was subsequently shown to be equally effective for the crystallization of DNA thanks to the commercial availability of affordable l-DNA sequences, with a variety of structural motifs now shown to form racemic crystals, enabling successful high-resolution structures to be determined (Doi *et al.*, 1993 ▸; Rypniewski *et al.*, 2006 ▸; Mandal *et al.*, 2014 ▸, 2016 ▸; Drozdzal *et al.*, 2016 ▸). Although a limited number of racemic DNA structures are available, some trends can be noted, including that racemates generally (although not always) crystallize in conditions under which the single enantiomer has been shown to crystallize, that equal or better quality crystallographic data are obtained from the racemates, and that the DNA conformations are the same in single-enantiomer and racemic DNA crystals grown under similar conditions.

Racemic DNA crystallography is thus of potential use to the structural biology field by enabling successful structure determination (particularly for challenging systems; Mandal *et al.*, 2016 ▸). In parallel, the combination of the physico-chemical properties of DNA (folding, stability, programmability and self-recognition) with access to all 230 space groups through the use of racemic DNA mixtures may give access to novel materials and/or self-assembled nano­structures. Indeed, beyond the variety of naturally occurring DNA structures (Neidle, 2021 ▸), DNA has also been studied extensively as a tool for the construction of precise self-assembled nano­materials and nano-objects with intriguing properties (Seeman, 2003 ▸, 2010 ▸; Rothemund, 2006 ▸; Aldaye *et al.*, 2008 ▸; Andersen *et al.*, 2009 ▸; McLaughlin *et al.*, 2011 ▸). In this vein, here we report two racemic crystal structures of the DNA sequence d(CCCGGG). This sequence forms highly ordered racemic crystals of an A-form duplex DNA, in contrast to the Z-form DNA observed in the crystal structure of the enantiopure equivalent (Gautham *et al.*, 1999 ▸). In addition, we observe the formation of racemic pseudo-helices within the crystal lattice, as well as the formation of large, water-filled channels. These racemic DNA structures and their unique, racemate-specific features thus suggest that racemic mixtures of DNA may enable the formation of solid-state organizations that are inaccessible to chiral samples.

## 2. Materials and methods

### 2.1. Crystallization and diffraction data collection

**Figure 1:**
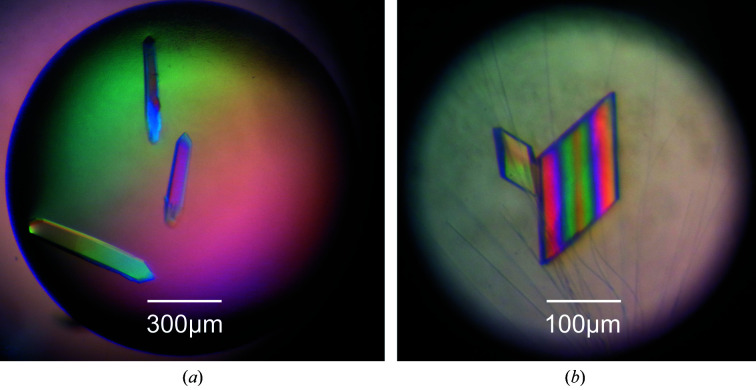
Crystals of d/l-d(CCCGGG) viewed under polarized light. X-ray diffraction measurements revealed that the crystals in (*a*) and (*b*) belonged to the achiral space groups 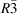 and *P*2_1_/*n*, respectively.

The l- and d-forms of the DNA sequence d(CCCGGG) were synthesized and purified by ChemGenes Corporation (USA). 2 m*M* DNA solutions of each enantiomer were prepared using ultrapure water. The enantiopure solutions were annealed at 353 K for 20 min and gradually cooled overnight to 293 K. After annealing, a racemic mixture was prepared by mixing the enantiopure solutions in an equimolar ratio. Crystallization experiments were carried out by the hanging-drop vapour-diffusion method at 293 K in hanging drops composed of 0.5 µl racemic DNA sample plus an equal volume of crystallization reagent: 2 *M* ammonium sulfate plus 100 m*M* Tris buffer pH 8.5 for crystal form 1 and 1.8 *M* ammonium sulfate plus 100 m*M* MES buffer pH 6.5 and 10 m*M* cobalt(II) chloride for crystal form 2 (Fig. 1 ▸).

For low-temperature diffraction measurements, crystals were mounted in nylon loops and vitrified in a stream of cold nitrogen gas at 150 K. Initial diffraction patterns confirmed the crystals to contain ordered DNA and revealed the presence of randomly oriented salt microcrystals. The DNA crystals were soaked briefly in 4 *M* trimethylamine *N*-oxide (TMO), which prevented the rapid formation of ammonium sulfate crystals and provided good cryoprotection (Mueller-Dieckmann *et al.*, 2011 ▸). X-ray diffraction data were measured using a microfocus rotating-anode Rigaku FR-X diffracto­meter with Cu *K*α radiation and a hybrid pixel detector (Dectris PILATUS 200K). Crystals 1 and 2 diffracted to resolutions of 2.48 and 2.80 Å, respectively. The diffraction data were indexed, integrated and scaled using the *XDS* package (Kabsch, 2010 ▸) and data statistics are summarized in Table 1 ▸.

### 2.2. Structure determination and refinement

Crystal 1 belonged to the achiral trigonal space group 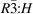 , with unit-cell parameters *a* = *b* = 105.41, *c* = 56.13 Å. The *E*-statistics distribution for this crystal indicates that the structure is centrosymmetric (Marsh, 1995 ▸). The structure was determined by molecular replacement (MR) using *Phaser* (McCoy *et al.*, 2007 ▸) from the *CCP*4 suite (Winn *et al.*, 2011 ▸). MR was successful using the crystal structure of d(CCCCGGGG)_2_ (PDB entry 1vt5; Haran *et al.*, 1987 ▸) as a search model, after failing when using the Z-DNA conformation of the single-enantiomer crystal of d-d(CCCGGG)_2_ (Gautham *et al.*, 1999 ▸). To build the MR model, the octa­nucleotide DNA strands were shortened to six residues by deletion of their first and eighth nucleotides. For MR, a multiple-copy search was carried out for two, three and four duplexes in the asymmetric unit. The MR solution, with a translation-function *Z* (TFZ) score of 9.1 and a log-likelihood gain (LLG) score of 118, revealed two independent d-DNA duplexes (hereafter named helices 1.1 and 1.2) in the asymmetric unit. The initial maps showed clearly resolved electron density for the duplex and allowed the unambiguous identification of the chirality of symmetry-related d- and l-enantiomers. The MR solution was refined using *phenix.refine* (Afonine *et al.*, 2012 ▸) in the *Phenix* suite (Liebschner *et al.*, 2019 ▸) with maximum-likelihood targets and NCS restraints (torsion angle). The restraint dictionary for nucleic acids was compiled by Parkinson *et al.* (1996 ▸). 10% of the unique reflections were used to calculate *R*_free_ (Brünger, 1992 ▸). Refinement of the coordinates was initially carried out with rigid-body refinement and individual coordinate refinement using simulated annealing. Subsequently, individual coordinate refinement was carried out in real space. Individual isotropic refinement of atomic displacement parameters (ADPs) was performed in combination with translation–libration–screw (TLS) refinement with the parameters generated in *phenix.refine* (Afonine *et al.*, 2012 ▸) using each DNA strand as a separate TLS group. After each refinement step, visual inspection of the model and the electron-density maps was carried out in *Coot* (Emsley *et al.*, 2010 ▸) using 2*F*_o_ − *F*_c_ and difference Fourier (*F*_o_ − *F*_c_) maps. The difference Fourier maps were contoured at the 5σ level in order to place ions or small molecules from crystallization reagents or cryoprotectants and at the 3σ level to identify water molecules. After refinement of the DNA strands, positive peaks were observed above 7σ in the difference Fourier map at two locations (Supplementary Fig. S1). In both locations, modelling and refinement was attempted with fully occupied water, sulfate ions and Tris buffer (from the crystallization reagent). In all cases the difference Fourier map showed residual electron density. Fitting of TMO (the cryoprotectant) into the electron density was carried out using *LigandFit* (Terwilliger *et al.*, 2007 ▸). Restraint generation and optimization for TMO was performed using *eLBOW* (Moriarty *et al.*, 2009 ▸) in the *Phenix* suite (Liebschner *et al.*, 2019 ▸. On the basis of ligand correlation score, peak height and refinement to reasonable *B* factors, we justify the assignment of these positive peaks as TMO. The fact that TMO was not co-crystallized and the inherent quality of the data presumably explain why the modelling of some TMO molecules remains poor. 49 water molecules were added throughout the various stages of refinement (Supplementary Fig. S1). The final *R* factor and *R*_free_ were 25.86% and 27.23%, respectively.

Crystal 2 belonged to the achiral monoclinic space group *P*2_1_/*n*, with unit-cell parameters *a* = 48.88, *b* = 41.43, *c* = 71.96 Å, β = 97.47°. The structure was solved by MR using a double helix (helix 1.1) from the trigonal form described above. For MR, a multiple-copy search was carried out for two, three and four duplexes in the asymmetric unit. The MR solution, with a translation-function *Z* (TFZ) score of 8.0 and a log-likelihood gain (LLG) score of 202, revealed three independent d-DNA duplexes (named helices 2.1, 2.2 and 2.3) in the asymmetric unit. The refinement protocol was similar to that implemented for the trigonal form described above. After refinement of the DNA strands, positive peaks were observed above 7σ in the difference Fourier *F*_o_ − *F*_c_ map at multiple locations (Supplementary Fig. S2). The two highest positive peaks were coordinated to the N^7^ atom of guanine G11 in helix 2.1 and in helix 2.2. Crystallographic studies reported that the cobalt(II) ion binds exclusively to N^7^ of guanine by coordination (Gao *et al.*, 1993 ▸). These two strong positive peaks were assumed to be, and were identified as, cobalt(II) ions from the crystallization reagent. The equivalent G11 in helix 2.3 was devoid of a strong positive peak. The average Co–N^7^ distance was 2.35 Å. The cobalt ions were refined with full occupancy, but due to the poor quality of the data the coordination sphere around them was not modelled. The remaining positive peaks were modelled as a chlorine ion and TMO. The density for the chlorine ion was broader than a water peak and was further away from the DNA atoms (3.5 Å from the N4 atom of cytosine). Modelling this peak with water gave residual density in the *F*_o_ − *F*_c_ map. Fitting and refinement of TMO molecules was as described above. Eight water molecules were added in the final stages of refinement (Supplementary Fig. S2). The final *R* factor and *R*_free_ were 27.95% and 31.82%, respectively. Further refinement did not lead to better convergence or to an improvement in the refinement statistics. One may note that the refinement *R* values were high compared with typical macromolecular structure depositions. This difference is at least in part inherent to centric data (Wilson, 1950 ▸).

The coordinates and structure factors were deposited in the Protein Data Bank (Berman *et al.*, 2000 ▸) with accession codes 6gn2 and 6gn3. Raw X-ray data for the two crystal structures have been deposited with Zenodo (at https://doi.org/10.5281/zenodo.6397509 for PDB entry 6gn2 and https://doi.org/10.5281/zenodo.6397557 for PDB entry 6gn3). The program 3*DNA* (Lu & Olson, 2003 ▸) was used to calculate helical parameters. Figures were prepared using *PyMOL* (DeLano, 2002 ▸). Root-mean-square deviation (r.m.s.d.) values were determined using *SUPERPOSE* (Krissinel & Henrick, 2004 ▸).

### 2.3. Circular-dichroism (CD) spectroscopy

CD spectra were recorded on a Jasco J-810 spectrometer using quartz cells with a 2 mm optical path length. Scans were acquired at 293 K as an average of four measurements over the range 200–320 nm, with a scanning speed of 50 nm min^−1^, a response time of 0.5 s, a data pitch of 0.2 nm and a bandwidth of 2 nm. 100 µ*M* DNA solutions were prepared in 2.0 *M* ammonium sulfate, 0.1 *M* Tris buffer pH 8.5 (crystallization condition 1), 1.8 *M* ammonium sulfate, 0.1 *M* MES buffer pH 6.5, 0.01 *M* cobalt(II) chloride (crystallization condition 2) and 1.0 *M* barium chloride, 50 m*M* sodium cacodylate pH 6.9, 0.001 *M* spermine·4HCl (the crystallization condition for the Z-form; Gautham *et al.*, 1999 ▸). Data for solutions of each crystallization condition were baseline-corrected for signal contributions due to buffer, salts and spermine·4HCl.

## 3. Results and discussion

**Figure 2:**
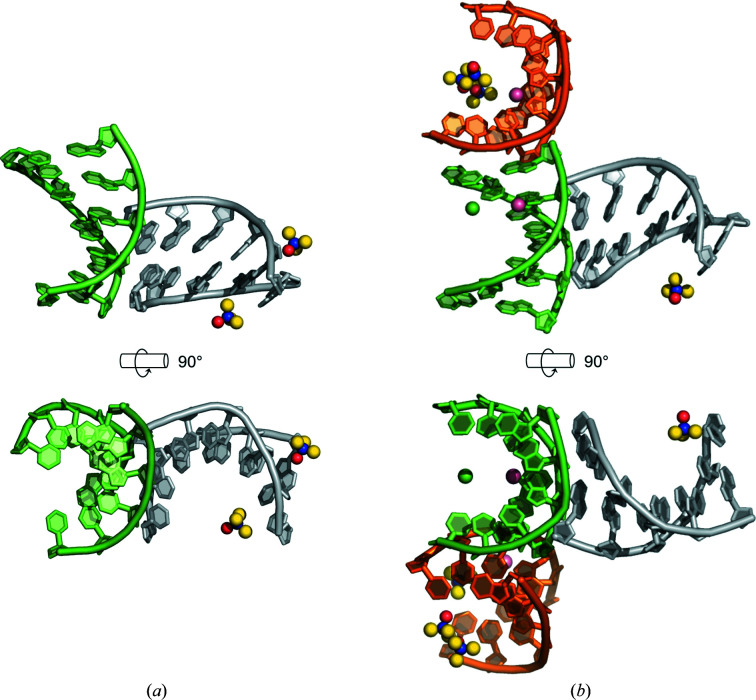
Asymmetric units of d/l-d(CCCGGG)_2_ in two racemic crystal forms: (*a*) 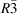 and (*b*) *P*2_1_/*n*. A-DNA helices are shown as cartoons. Trimethylamine *N*-oxide, cobalt ions (pink) and chlorine ions (green) are shown as spheres. Bound waters are omitted for clarity.

Previously, crystallization of the enantiopure d-d(CCCGGG) sequence was reported by Gautham and coworkers using the hanging-drop method at a high salt concentration (1 *M* barium chloride plus 50 m*M* sodium cacodylate buffer pH 6.9, 1 m*M* spermine; Gautham *et al.*, 1999 ▸). Despite the lack of multiple alternating pyrimidine–purine base steps, this enantiopure sequence was shown by Gautham and coworkers to form a Z-DNA duplex. We used similar conditions to attempt to crystallize the racemic mixture [*i.e.* d/l-d(CCCGGG)]; however, we were unsuccessful in obtaining crystals. We next turned to a sparse-matrix crystallization screening approach, which successfully identified two related conditions suitable for crystal growth. X-ray diffraction analyses of the two crystal forms indicated that the crystals belonged to the achiral space groups 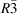 and *P*2_1_/*n*, in line with previous findings describing the tendency of racemic DNA mixtures to form racemic crystals rather than conglomerates (Mandal *et al.*, 2014 ▸). Both crystal structures were solved by molecular replacement, with the resulting refined electron-density maps being of sufficiently high quality to allow the chirality of the DNA strands to be unambiguously determined. Surprisingly, both crystal forms of d/l-d(CCCGGG)_2_ reported here revealed DNA structures adopting A-type duplex conformations (Fig. 2 ▸). Analysis of the duplex geometry reveals parameters that are all in line with expectations for A-DNA, namely a rise per residue of ∼2.8–3.0 Å, a base-pair inclination of ∼9–13° and a preference for C3′-*endo* sugar puckers (Supplementary Table S2). To our knowledge, this is the first report of a racemic crystal structure of A-form DNA.

**Figure 3:**
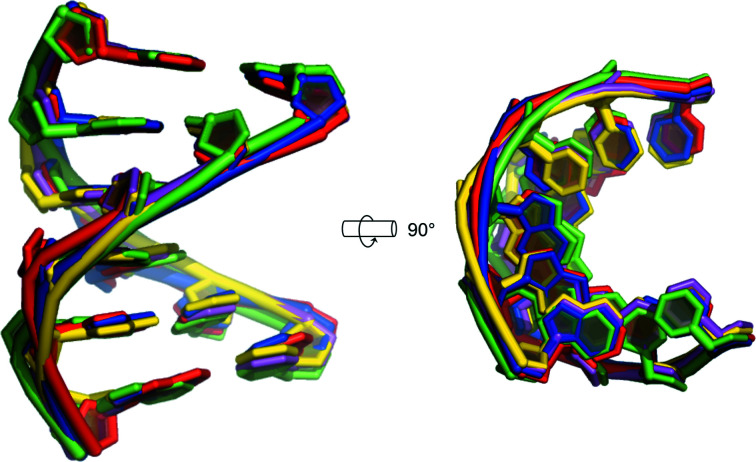
Superposition of all d-DNA molecules found in the two structures of d/l-d(CCCGGG)_2_. Helices 1.1 and 1.2 (space group 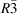 ) are coloured red and green, respectively. Helices 2.1, 2.2 and 2.3 (space group *P*2_1_/*n*) are coloured blue, yellow and magenta, respectively. R.m.s.d.s are reported in Supplementary Table S1.

The adoption of A-form duplexes by the racemic form of this sequence was unexpected, as previous spectroscopic studies of the equivalent enantiopure sequence [*i.e.* d-d(CCCGGG)] revealed B-type duplex DNA, albeit with non­standard deoxyribose conformations and internucleotide stacking geometry (Wolk *et al.*, 1989 ▸). In addition, as stated above, previous crystallographic studies of the enantiopure d(CCCGGG) sequence revealed an unusual Z-form conformation with nonstandard Z-form geometry (Gautham *et al.*, 1999 ▸). The two strands in this duplex had different backbone conformations: the phosphate groups of one strand had a zigzag arrangement, while those in the other strand formed a smooth continuous helix. A study prior to this had also revealed the enantiopure d(CCCGGG) sequence to adopt a conformation intermediate between A-DNA and B-DNA when bound to the antibiotic nogalamycin (Cruse *et al.*, 1996 ▸). We measured CD spectra of d-d(CCCGGG) under the conditions that gave rise to the two A-form and the Z-form crystals (Supplementary Fig. S3). Under all conditions, the spectra had the characteristics of B-form DNA, with a negative band near 240 nm and a positive band above 260 nm. The absence of a strong negative band near 290 nm suggested no strong contribution from a Z-form, and the absence of a negative band below 220 nm indicated that the A-form was not prevalent (Kypr *et al.*, 2009 ▸). Thus, both the racemic A-form and enantiopure Z-form appear to reflect effects that occur only in the solid state, a phenomenon that has been proposed for other nucleic acid structures (Sheehan *et al.*, 2019 ▸). The racemic A-form duplex crystal structures that we report here therefore add to the array of conformations adopted by this DNA sequence, providing further evidence of the considerable structural polymorphism of this motif. Perhaps paradoxically, despite this apparent structural polymorphism, the five independent A-type duplexes present within these two crystal structures reveal a remarkable degree of structural similarity, as indicated by structural alignments (Fig. 3 ▸ and Supplementary Table S1), despite the differences in space group and crystal packing.

**Figure 4:**
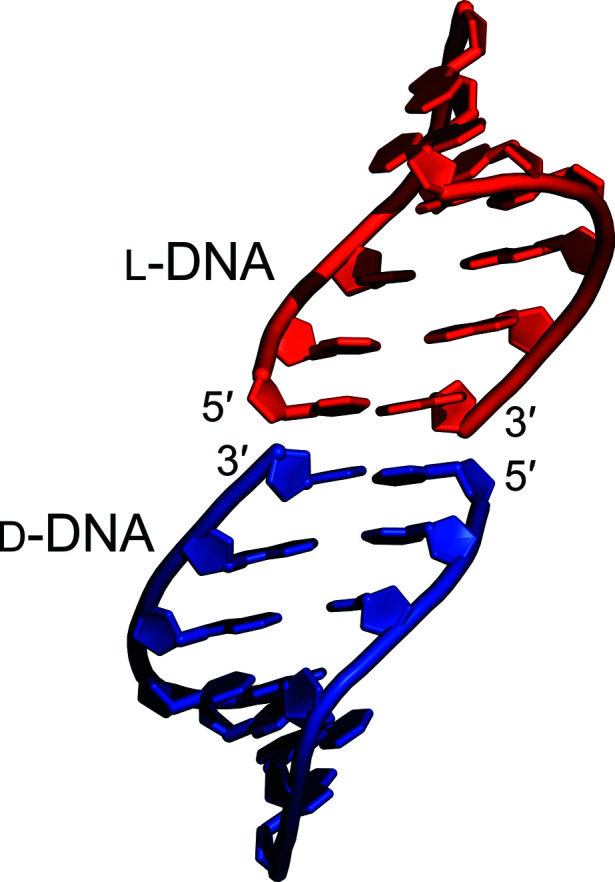
Packing of l- and d-form A-DNA duplexes into heterochiral pseudo­helices within the lattice of crystal form 1. An almost identical pattern is observed in crystal form 2.

Analysis of the crystal-packing interactions reveals further surprising features, with the lattices of both crystal forms reported here showing both homochiral and heterochiral pseudo-continuous helices (Fig. 4 ▸, Supplementary Figs. S6 and S7), as well as linear water-filled channels (Supplementary Fig. S8). Heterochiral pseudo-continuous helices are helices in which d-DNA duplexes and l-DNA duplexes stack end-to-end in an alternating manner, with an inversion of the helix handedness at every helix–helix contact. Previous reports of racemic DNA sequences have revealed a predominance of homochiral pseudo-helices within the crystal lattices (Mandal *et al.*, 2014 ▸; Supplementary Fig. S4), with few reports of heterochiral pseudo-helical packing (Doi *et al.*, 1993 ▸; Rypniewski *et al.*, 2006 ▸; Supplementary Fig. S5) and none for A-form DNA. Thus, the racemic d/l-d(CCCGGG) sequence studied here reveals packing interactions that have not previously been observed, suggesting that racemic mixtures of DNA sequences may generate assemblies and higher-order structures that are not accessible to equivalent enantiopure systems.

As mentioned in Section 1, artificial chiral DNA systems have been used to engineer nano-architectures such as nanocontainers (Sun *et al.*, 2014 ▸) and DNA–protein hybrid shapes (Praetorius & Dietz, 2017 ▸). Rationally engineered DNA crystals have also been produced using l-DNA in order to confer them with resistance towards nuclease degradation (Simmons *et al.*, 2017 ▸). Speculation that racemic DNA may be of benefit to such endeavours may not be entirely fanciful.

## 4. Conclusions

Here, we have reported two racemic crystal structures of the DNA sequence d(CCCGGG) revealing A-type duplexes, in contrast to previous structural descriptions of the enantiopure form of this sequence. Why does the racemic mixture of this hexameric DNA sequence adopt a different conformation in the solid state compared with the enantiopure equivalent? We cannot currently answer this question, but it appears that neither reflects the prevalent species in solution. Crystal-packing forces are thus likely to be at the origin of the lattices that are observed. The conditions required to capture the Z-form of the racemic d(CCCGGG) mixture in the solid state have yet to be discovered. However, what can be concluded with some confidence is that a racemic crystallography approach has allowed us to identify a structural form of a DNA sequence that has not previously been observed for the enantiopure equivalent. Our results add to other established uses of l-DNA including, for example, as interacting molecules with proteins (An *et al.*, 2020 ▸; Vater & Klussmann, 2015 ▸). Considering the potential for such systems to generate novel solid-state self-assemblies and nanomaterials, we believe that further investigation may be warranted.

## 5. Related literature

The following reference is cited in the supporting information for this article: Altona & Sundaralingam (1972 ▸).

## Supplementary Material

PDB reference: d(CCCGGG), 6gn2

PDB reference: 6gn3

Raw X-ray data for PDB entry 6gn2.: https://doi.org/10.5281/zenodo.6397509

Raw X-ray data for PDB entry 6gn3.: https://doi.org/10.5281/zenodo.6397557

Supplementary Tables and Figures. DOI: 10.1107/S2059798322003928/di5054sup1.pdf

## Figures and Tables

**Table 1:** Summary of X-ray diffraction data and refinement Values in parentheses are for the outer shell.

Crystal form	1	2
Data collection		
Space group	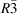	*P*2_1_/*n*
*a*, *b*, *c* (Å)	105.41, 105.41, 56.13	48.88, 41.43, 71.96
α, β, γ (°)	90.00, 90.00, 120.00	90.00, 97.47, 90.00
Resolution range (Å)	23.91–2.48 (2.57–2.48)	31.49–2.80 (2.90–2.80)
Total reflections	15782 (1279)	13035 (1332)
Unique reflections	8101 (729)	6839 (685)
Multiplicity	1.9 (1.8)	1.9 (1.9)
Completeness	0.97 (0.89)	0.99 (0.99)
Mean *I*/σ(*I*)	10.71 (2.02)	11.19 (2.47)
*R*_merge_ (%)	5.74 (42.49)	7.30 (33.93)
*R*_meas_ (%)	8.11 (60.09)	10.32 (47.99)
CC_1/2_	0.997 (0.926)	0.990 (0.924)
Wilson *B* factor (Å^2^)	49.67	49.82
Refinement		
No. of reflections	8101 (725)	6839 (685)
*R*_work_ (%)	24.96 (36.60)	27.95 (41.87)
*R*_free_ (%)	26.83 (42.57)	31.82 (52.96)
No. of non-H atoms		
Total	539	751
DNA	480	720
Ions	—	2 Co, 1 Cl
TMO	10	20
Water	49	8
R.m.s.d., bond lengths (Å)	0.003	0.007
R.m.s.d., angles (°)	0.39	0.56
Mean *B* factor (Å^2^)	34.27	49.17
PDB code	6gn2	6gn3
