# Supplementary material for: Racemic crystal structures of A-DNA duplexes
Source: Acta Crystallogr D Struct Biol. 2022 May 9;78(Pt 6):709–15. doi: 10.1107/S2059798322003928 (PMC9159285; doi:10.1107/S2059798322003928)
Supplement: Supplementary file 1 [file d-78-00709-sup1.pdf]

STRUCTURAL BIOLOGY

Volume 78 (2022)

Supporting information for article:

Racemic crystal structures of A-DNA duplexes

Pradeep K. Mandal, Gavin W. Collie, Brice Kauffmann and Ivan Huc

Table S1 R.m.s.d. values (in Å) of the five unique A-DNA helices superposed based on calculations using *Superpose* (Krissinel and Henrick, 2004).

Superpose calculations performed for all atoms of residues 1 to 6 and 7 to 12 of the two strands in each helix.

Helices	$R\bar{3}$- Helix-1	$R\bar{3}$- Helix-2	$P2_1$/n- Helix-1	$P2_1$/n- Helix-2	$P2_1$/n- Helix-3
$R\bar{3}$- Helix-1	-	1.24	0.60	0.99	0.88
$R\bar{3}$- Helix-2	1.24	-	1.09	1.11	0.82
$P2_1$/n- Helix-1	0.60	1.09	-	0.79	0.71
$P2_1$/n- Helix-2	0.99	1.11	0.79	-	0.50
$P2_1$/n- Helix-3	0.88	0.82	0.71	0.50	-

Table S2 The pseudorotation parameters (Altona and Sundaralingam, 1972) of the deoxyriboses in the five unique A-DNA helices based on calculations using the program *3DNA* (Lu and Olson, 2003).

Helices	Nucleoside	T_M (°)	P (°)	Sugar pucker	Helices	Nucleoside	T_M (°)	P (°)	Sugar pucker
$R\bar{3}$-Helix-1	C^{1}	40.2	13.4	C3’-*endo*	$P2_1$/n-Helix-1	C^{7}	38.9	17.1	C3’-*endo*
	C^{2}	38.8	15.3	C3’-*endo*		C^{8}	38.1	20.5	C3’-*endo*
	C^{3}	42.5	21.2	C3’-*endo*		C^{9}	40.4	18.7	C3’-*endo*
	G^{4}	43.9	21.0	C3’-*endo*		G^{10}	40.6	14.6	C3’-*endo*
	G^{5}	38.3	4.3	C3’-*endo*		G^{11}	44.1	22.1	C3’-*endo*
	G^{6}	39.0	19.1	C3’-*endo*		G^{12}	37.9	21.7	C3’-*endo*
	C^{7}	39.5	15.1	C3’-*endo*	$P2_1$/n-Helix-2	C^{1}	37.0	16.0	C3’-*endo*
	C^{8}	39.5	15.0	C3’-*endo*		C^{2}	35.4	21.8	C3’-*endo*
	C^{9}	38.8	17.8	C3’-*endo*		C^{3}	39.2	19.4	C3’-*endo*
	G^{10}	39.7	19.7	C3’-*endo*		G^{4}	38.5	14.3	C3’-*endo*
	G^{11}	43.2	8.3	C3’-*endo*		G^{5}	41.0	17.7	C3’-*endo*
	G^{12}	41.0	21.4	C3’-*endo*		G^{6}	37.4	21.5	C3’-*endo*
$R\bar{3}$-Helix-2	C^{1}	39.2	14.1	C3’-*endo*		C^{7}	38.5	13.5	C3’-*endo*
	C^{2}	40.4	17.7	C3’-*endo*		C^{8}	38.6	21.7	C3’-*endo*
	C^{3}	41.6	19.6	C3’-*endo*		C^{9}	42.6	19.3	C3’-*endo*
	G^{4}	41.9	19.8	C3’-*endo*		G^{10}	41.9	16.0	C3’-*endo*
	G^{5}	42.2	8.5	C3’-*endo*		G^{11}	40.9	21.1	C3’-*endo*
	G^{6}	39.3	22.5	C3’-*endo*		G^{12}	33.2	20.6	C3’-*endo*
	C^{7}	39.2	14.5	C3’-*endo*	$P2_1$/n-Helix-3	C^{1}	39.1	13.1	C3’-*endo*
	C^{8}	38.8	14.9	C3’-*endo*		C^{2}	37.1	19.7	C3’-*endo*
	C^{9}	40.5	18.7	C3’-*endo*		C^{3}	42.2	17.8	C3’-*endo*
	G^{10}	39.6	20.2	C3’-*endo*		G^{4}	43.7	16.3	C3’-*endo*
	G^{11}	40.6	7.3	C3’-*endo*		G^{5}	44.7	21.6	C3’-*endo*
	G^{12}	38.6	17.7	C3’-*endo*		G^{6}	39.7	25	C3’-*endo*
$P2_1$/n-Helix-1	C^{1}	38.8	16.6	C3’-*endo*		C^{7}	38.3	15.0	C3’-*endo*
	C^{2}	36.8	21.6	C3’-*endo*		C^{8}	38.4	22.1	C3’-*endo*
	C^{3}	40.4	19.3	C3’-*endo*		C^{9}	44.1	18.4	C3’-*endo*
	G^{4}	43.0	13.1	C3’-*endo*		G^{10}	44.6	13.5	C3’-*endo*
	G^{5}	42.0	19.2	C3’-*endo*		G^{11}	43.4	19.3	C3’-*endo*
	G^{6}	36.2	19.7	C3’-*endo*		G^{12}	37.0	25.1	C3’-*endo*

T_M: amplitutde of pseudorotation of the sugar ring given in degrees.

P: phase angle of pseudorotation of the sugar ring given in degrees.

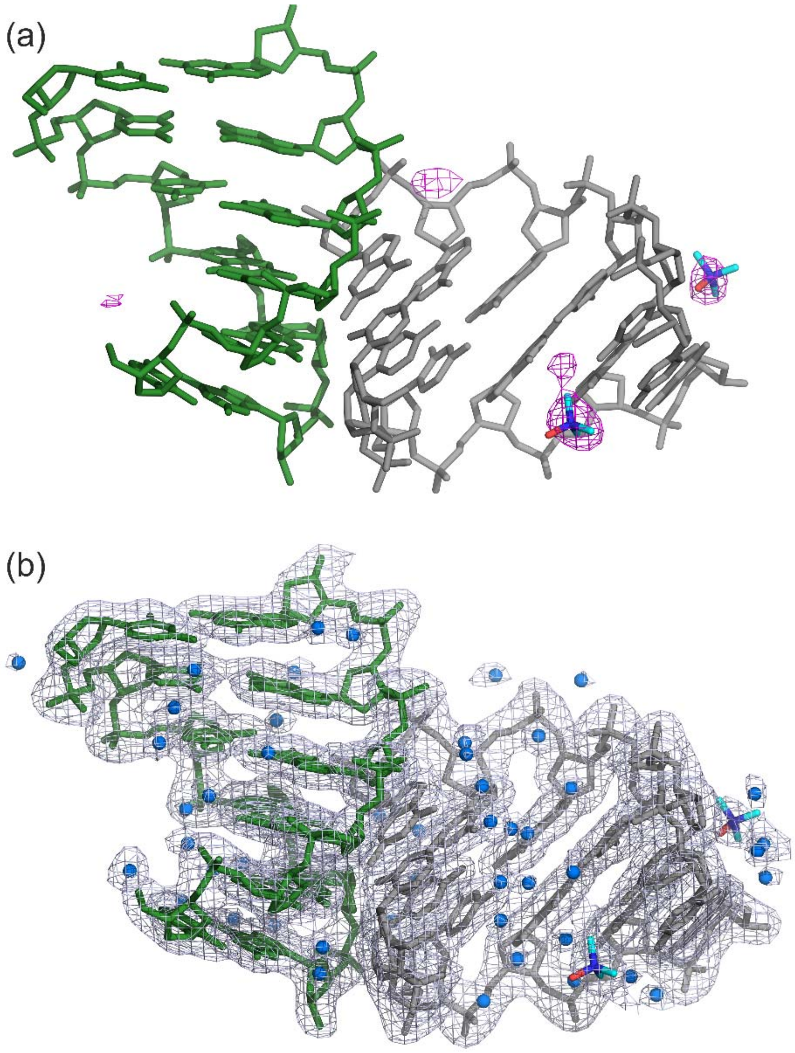

Figure S1 Electron density maps superimposed on the crystal structure of L/D- d(CCCGGG)$_2$ in space group $R\overline{3}$. A-DNA helices and trimethylamine N-oxide shown as sticks. (a) Sigma-weighted difference Fourier (F_o-F_c) map (magenta mesh) contoured at 5 σ level. Water molecules are omitted for clarity. (b) Sigma-weighted 2F_o-F_c map (grey mesh) contoured at 1 σ level. Water molecules shown as blue spheres.

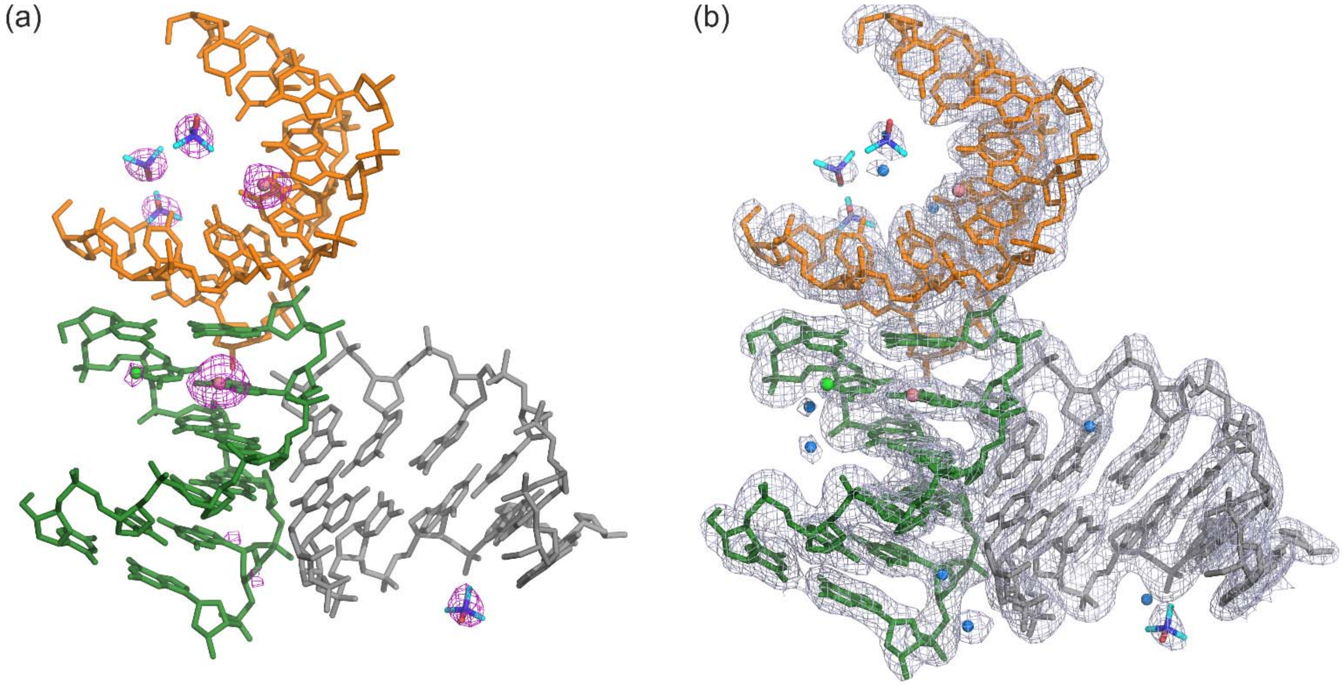

Figure S2 Electron density maps superimposed on the crystal structure of D/L-d(CCCGGG)$_2$ in space group $P2_1$/n. A-DNA helices and trimethylamine N-oxide shown as sticks. (a) Sigma-weighted difference Fourier (F_o-F_c) map (magenta mesh) contoured at 5 σ level. Water molecules are omitted for clarity. (b) Sigma-weighted $2F_o$-F_c map (grey mesh) contoured at 1 σ level. Cobalt ions (pink), Chlorine ion (green) and water molecules (blue) shown as spheres.

Figure S3 CD spectra of 100μM L-d(CCCGGG) measured at 293 K in 2.0 M ammonium sulfate, and 0.1 M TRIS buffer (pH 8.5) (crystallization condition 1); 1.8 M ammonium sulfate, 0.1 M MES buffer (pH 6.5) and 0.01 M cobalt (II) chloride (crystallization condition 2)and 1.0 M barium chloride, 50 mM sodium cacodylate (pH 6.9), and 0.001 M spermine 4 HCl (crystallization condition of Z-form).

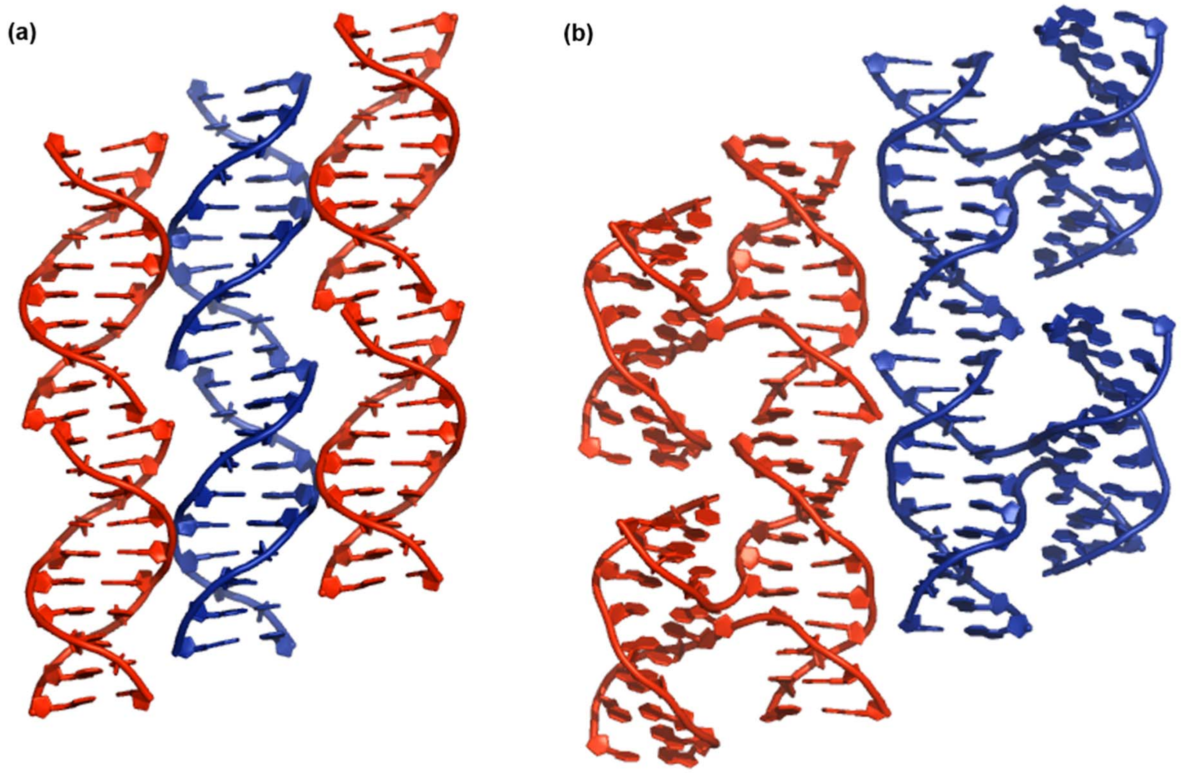

Figure S4 Homo-chiral pseudo-continuous helical stacking observed for D/L-d(CCGGTACCGG) crystallized as (a) B-DNA duplexes (PDB ID 4R49) and (b) four-way Holliday junctions with B-DNA arms (PDB ID 4R48). Left and right handed helices are shown in red and blue, respectively.

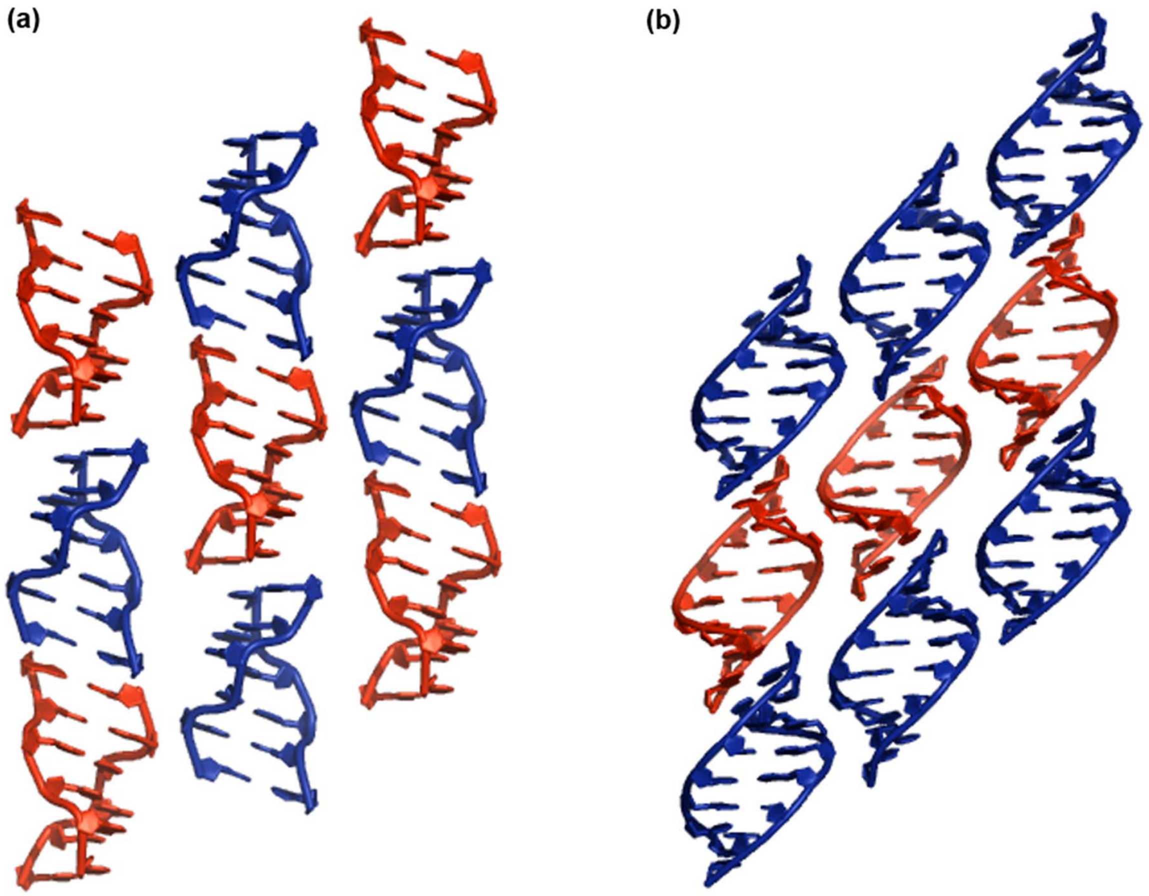

Figure S5 Hetero-chiral pseudo-continuous helical stacking observed for (a) D/L-d(CGCGCG)$_2$ crystallized as Z-DNA duplexes (PDB ID 1VTU) and (b) D/L-r(CUGGGCGG).r(CCGCCUGG) crystallized as A-RNA duplexes (PDB ID 2GQ6). Left and right handed helices are shown in red and blue, respectively.

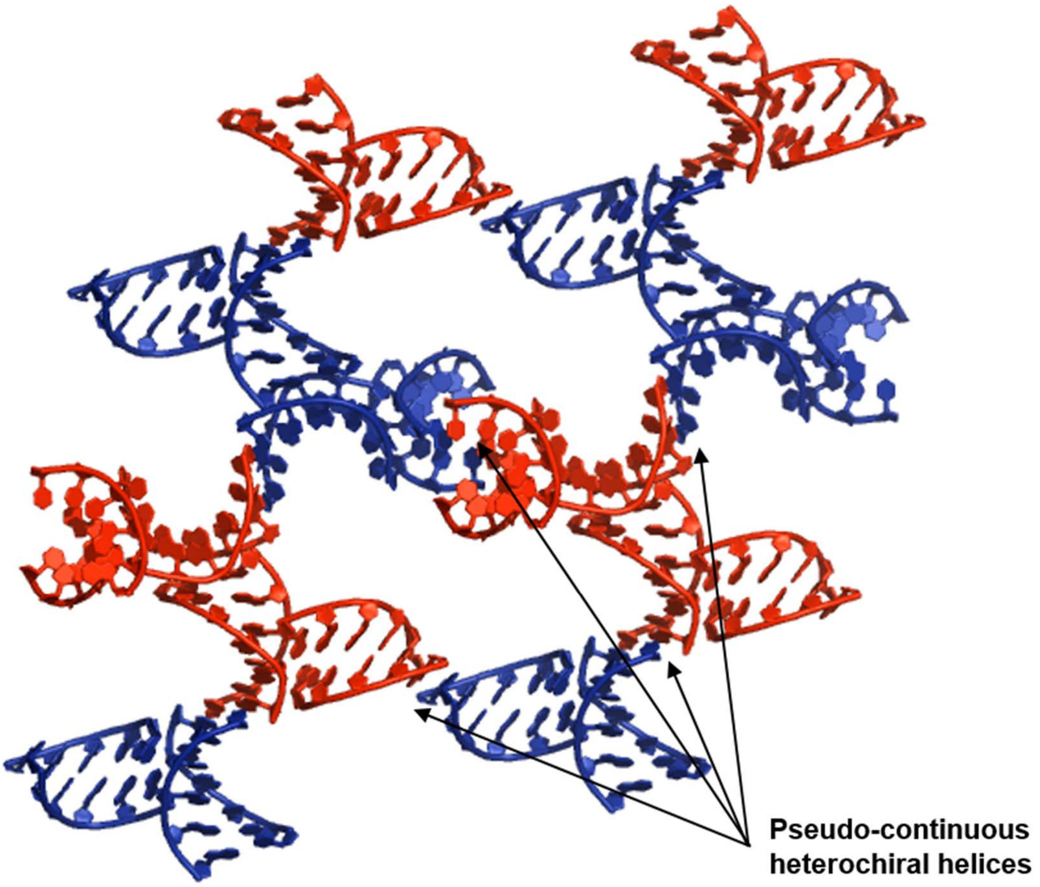

Figure S6 Hetero-chiral pseudo-continuous helical stacking observed for D/L-d(CCCGGG)$_2$ crystallized as A-DNA duplexes (PDB ID 6GN2) in the space group $R\bar{3}$. Left and right handed helices are shown in red and blue, respectively.

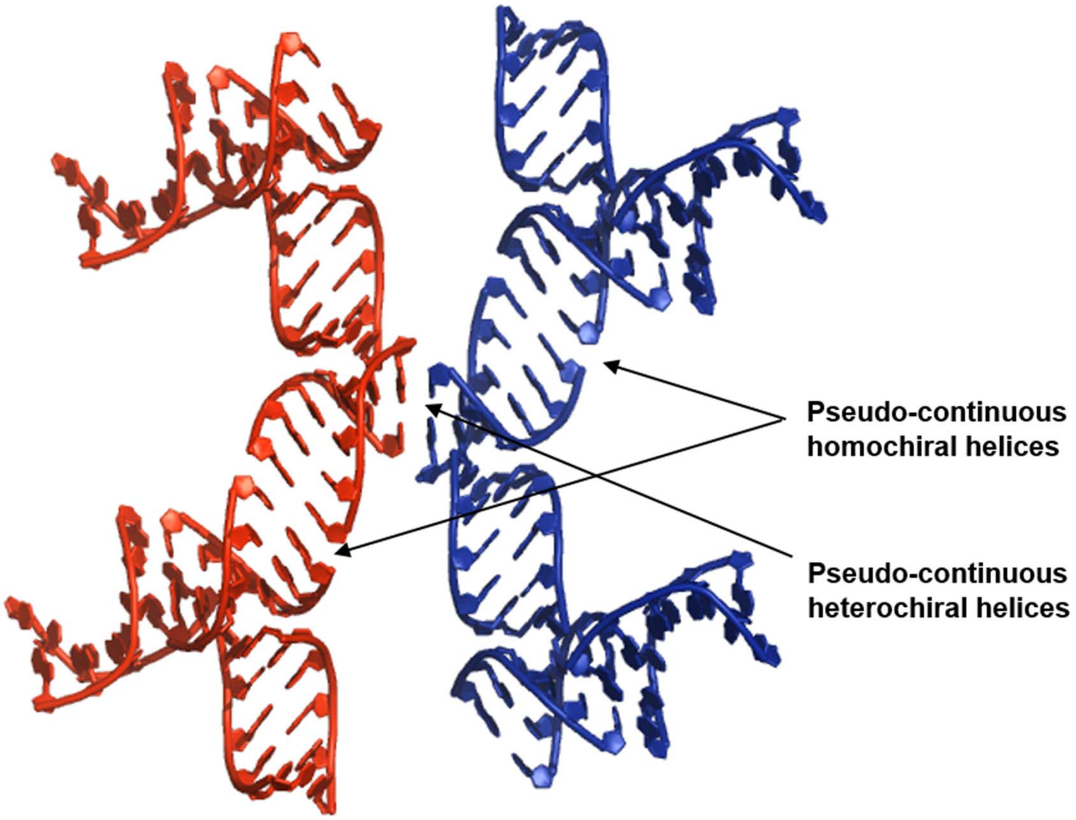

Figure S7 Homo- and hetero-chiral pseudo-continuous helical stacking observed for D/L-d(CCCGGG)$_2$ crystallized as A-DNA duplexes (PDB ID 6GN3) in the space group P2$_1$/n. Left and right handed helices are shown in red and blue, respectively. Terminal base pair and minor-groove interactions between neighbouring helices are chiral-selective.

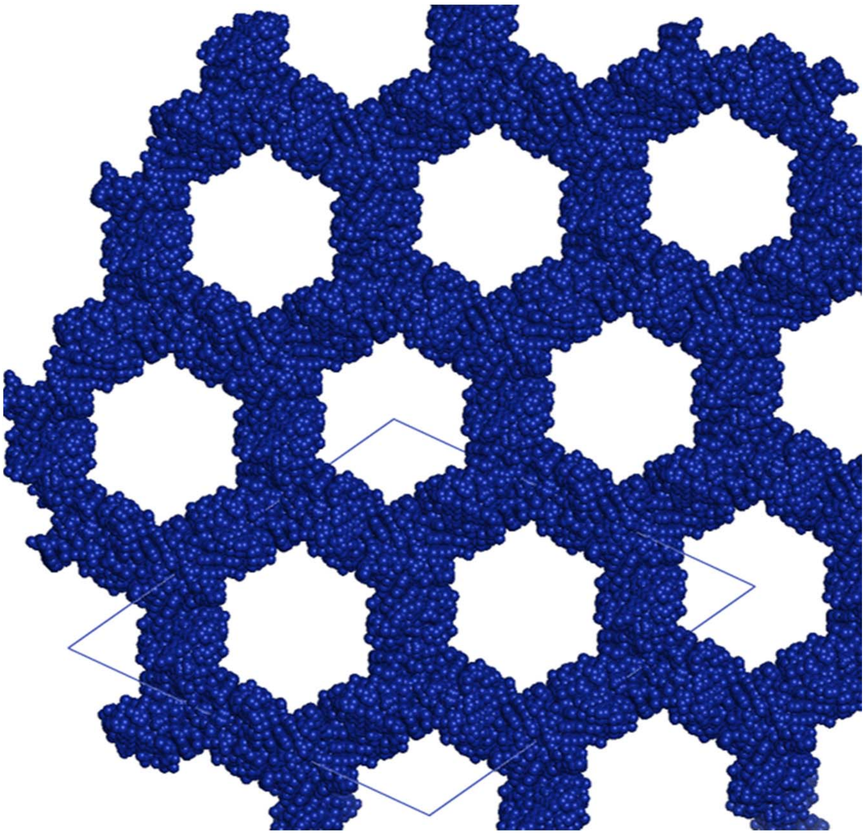

Figure S8 The packing of L/D-d(CCCGGG)$_2$ reveals linear hexagonal solvent channels. DNA molecules are shown as blue spheres.
